# Posterior Left pericardiotomy for the prevention of postoperative Atrial fibrillation after Cardiac Surgery (PALACS): study protocol for a randomized controlled trial

**DOI:** 10.1186/s13063-017-2334-4

**Published:** 2017-12-13

**Authors:** Ahmed A. Abouarab, Jeremy R. Leonard, Lucas B. Ohmes, Christopher Lau, Lisa Q. Rong, Natalia S. Ivascu, Kane O. Pryor, Monica Munjal, Filippo Crea, Massimo Massetti, Tommaso Sanna, Leonard N. Girardi, Mario Gaudino

**Affiliations:** 1 Department of Cardiovascular Sciences, Catholic University, Rome, Italy; 2 Department of Cardiothoracic Surgery, Weill Cornell Medicine, 525 East 68th St, New York, NY 10065 USA

**Keywords:** Atrial fibrillation, Arrhythmia, Postoperative atrial fibrillation, Cardiac surgery, Postoperative arrhythmia

## Abstract

**Background:**

Postoperative atrial fibrillation (POAF) is a common complication following cardiac surgery. POAF is associated with increased morbidity and hospital costs. We herein describe the protocol for a randomized controlled trial to determine if performing a posterior left pericardiotomy prevents POAF after cardiac surgery.

**Methods/design:**

All patients submitted to cardiac surgery at our institution will be screened for inclusion into the study. The study will consist of two parallel arms with random allocation between groups to either receive a posterior left pericardiotomy or serve as a control. Masking will be done in a single-blinded fashion to the patient. Patients will be continuously monitored postoperatively for the occurrence of atrial fibrillation until discharge. At the follow-up clinic visit (15–30 days after surgery), the primary endpoint (atrial fibrillation) and other secondary endpoints, such as pleural or pericardial effusion, will be assessed. A total sample size of 350 subjects will be recruited.

**Discussion:**

POAF is associated with increased morbidity, prolonged hospital stay, and increased costs after cardiac surgery. Several strategies aimed at reducing the incidence of POAF have been investigated, including beta-blockers, amiodarone, and statins, all with suboptimal results. Posterior left pericardiotomy has been associated with a reduction of POAF in previous series. However, these studies had limited sample sizes and suboptimal methodology, so that the efficacy of posterior pericardiotomy in preventing POAF remains to be definitively proven. Our randomized trial aims to determine the effect of a posterior left pericardiotomy on the incidence of POAF.

**Trial registration:**

ClinicalTrials.gov, ID: NCT02875405, protocol record 1502015867. Registered on July 2016.

**Electronic supplementary material:**

The online version of this article (doi:10.1186/s13063-017-2334-4) contains supplementary material, which is available to authorized users.

## Background

Postoperative atrial fibrillation (POAF) is a common complication following cardiac surgery with an incidence of 30–40% [1, 2]. POAF has been associated with stroke, systemic embolism, and cardiac failure. Its detection mandates additional treatment with varying combinations of drugs to control cardiac rate and/or rhythm, anticoagulation, and/or electrical cardioversion; each of which have side effects that cannot be ignored. As a result, POAF prolongs hospital stay and increases hospital expenditure [1]. Several strategies aimed at reducing the incidence of POAF have been investigated, including treatment with beta-blockers, amiodarone, and statins, all with unsatisfactory results [1, 3–5]. In a few studies, performing a posterior left pericardiotomy has been associated with a reduction in the incidence of POAF by allowing drainage of blood and fluid from the posterior pericardial space [6–8]. Performing a posterior left pericardiotomy is theorized to prevent POAF by allowing the posterior pericardial space to drain into the left side of the chest and be evacuated by a chest tube. This prevents blood and fluid from accumulating behind the left atrium, causing atrial irritation which can lead to POAF. However, previous studies are associated with some limitations such as: sample size, inclusion/exclusion criteria, randomization procedure, and suboptimal electrocardiographic monitoring strategies. Moreover, posterior left pericardiotomy requires additional operative time and can be associated with procedure-specific complications such as an increased incidence of left-sided pleural effusion. As a result, current evidence on posterior pericardiotomy has failed to translate into changes to clinical practice.

The primary objective of the present prospective, randomized, controlled study is to assess whether performing a posterior left pericardiotomy during open cardiac surgery procedures (coronary artery bypass grafting (CABG), aortic valve replacement, interventions to the ascending aorta or their combination) results in a reduction in the incidence of POAF.

The secondary objectives of this study are to assess:

- The time spent in atrial fibrillation
- The duration of the hospitalization
- Safety measures such as: pericardial and left-sided pleural effusion as a complication of this procedure, death, and major adverse events (MAE)

This study contributes to the cardiovascular community’s understanding of POAF and has the potential to offer an alternative treatment option that mitigates the need for anticoagulation and antiarrhythmic drugs along with their associated side effects. This is especially important for patients who have contraindications to either anticoagulation or antiarrhythmic therapy.

## Methods and design

The study was designed according to the Recommendations for Interventional Trials (SPIRIT) guidelines (see Additional file 1: SPIRIT Checklist, Figure S1).

### Methods: participants, interventions, outcomes

#### Study setting

The Department of Cardiothoracic Surgery at Weill Cornell Medicine/New York Presbyterian Hospital.

#### Eligibility criteria

Included subjects will be patients undergoing open cardiac surgery for interventions on the coronary arteries, the aortic valve, and/or the ascending aorta who have no previous history of atrial fibrillation. Exclusion criteria are as follows: pre-operative non-sinus rhythm, history of previous atrial arrhythmia of any type, reoperation, mitral or tricuspid valve disease, surgery of the descending thoracic or thoraco-abdominal aorta, need for hypothermic circulatory arrest, off-pump operations, urgent or emergent presentation, disease of the left-sided pleura or previous left-sided instrumentation, non-cardiac-related comorbid contraindications to surgery, and chest deformity of any kind.

#### Interventions

In the intervention group, a posterior left pericardiotomy will be performed according to technique described in the surgical literature [9]. In brief, if not already entered, the left pleural space will be entered through the pre-existing median sternotomy. A 4-cm incision will be made posterior to the phrenic nerve in a parallel and longitudinal fashion extending from the left inferior pulmonary vein to the diaphragm. We will use the tip of the mediastinal tube that is placed in all cardiac surgery patients (instead of an additional chest tube) for drainage of the left-sided pleura so that patients of the treatment group will not have a higher level of postoperative pain or discomfort. The estimated additional surgery time is 15 min.

#### Concomitant care

**Fig. 1:**
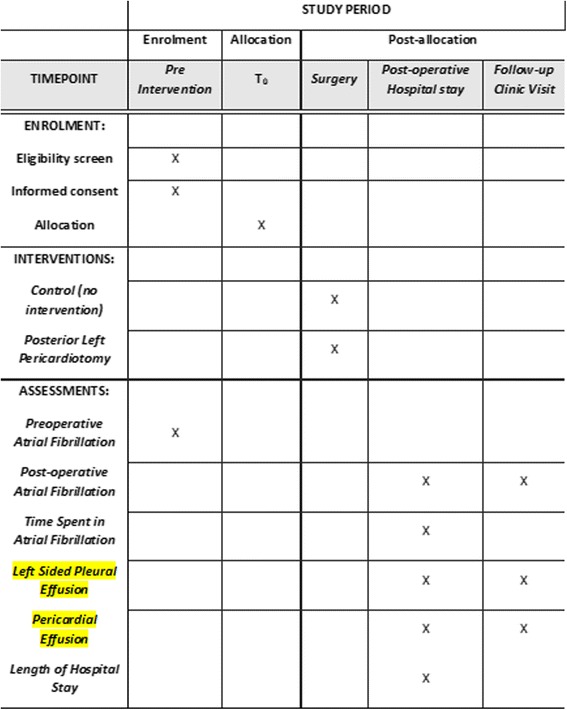
Standard Protocol Items: Recommendations for Interventional Trials (SPIRIT) Figure

According to our current practice, patients who were taking beta-blockers pre-operatively will continue taking the beta-blocker up to the time of surgery. After surgery, all patients are given beta-blockers per os starting on postoperative day 1, except those who are bradycardic (heart rate < 65 beats per minute (bpm)), require epicardial pacing, have AV block, or are receiving beta agonists, such as epinephrine or dobutamine. In those cases, when the patient’s intrinsic heart rate increases to > 65 bpm, AV block resolves, or epinephrine is discontinued, beta-blockers are then initiated. Any inotropic agents that may contribute to development of atrial arrhythmias are weaned and discontinued as rapidly as the patients’ hemodynamic parameters will allow. This protocol will be applied in both groups in the same way. All patients will have an immediate postoperative chest radiograph (CXR) followed by a daily morning CXR for the entire hospitalization. Patients who are discharged with a moderate or larger pleural effusion will have another CXR when they are evaluated in clinic 2–3 weeks after discharge. A transthoracic echocardiogram will be performed before discharge and at the follow-up visit, to evaluate for pericardial effusion (SPIRIT Figure, Fig. 1). In case of evidence of moderate-to-severe pericardial effusion, further echocardiography or computer tomography evaluation and drainage will be performed when necessary.

#### Outcomes

The primary outcome of the study is the occurrence of POAF, defined as the occurrence of an irregularly irregular heart rhythm, without detectable P waves, lasting more than 30 s observed during the hospital stay. Patients will be monitored continuously during the entire hospital stay with the Philips Intellivue MP70 patient monitor (Philips, Andover, MA, USA) and alarm strips will be collected for analysis and POAF adjudication. A standard 12-lead electrocardiogram (EKG) will be recorded on a daily basis and collected for analysis and POAF adjudication. Additional 12-lead standard EKGs ordered at the discretion of the caring physician will be collected for analysis and POAF adjudication. An independent committee blinded to group assignment will adjudicate episodes of POAF. The secondary outcome measures of the study will include: (1) time spent in atrial fibrillation, defined as the time from the first evidence of atrial fibrillation to the first evidence of sinus rhythm restoration on cardiac monitoring strips or standard EKG, (2) duration of hospitalization, (3) antiarrhythmic drug use, and (4) need for electrical cardioversion. Safety outcomes will be (1) incidence of left pleural effusion, (2) incidence of pericardial effusion, (3) MAE, and (4) death.

#### Participant timeline

**Fig. 2:**
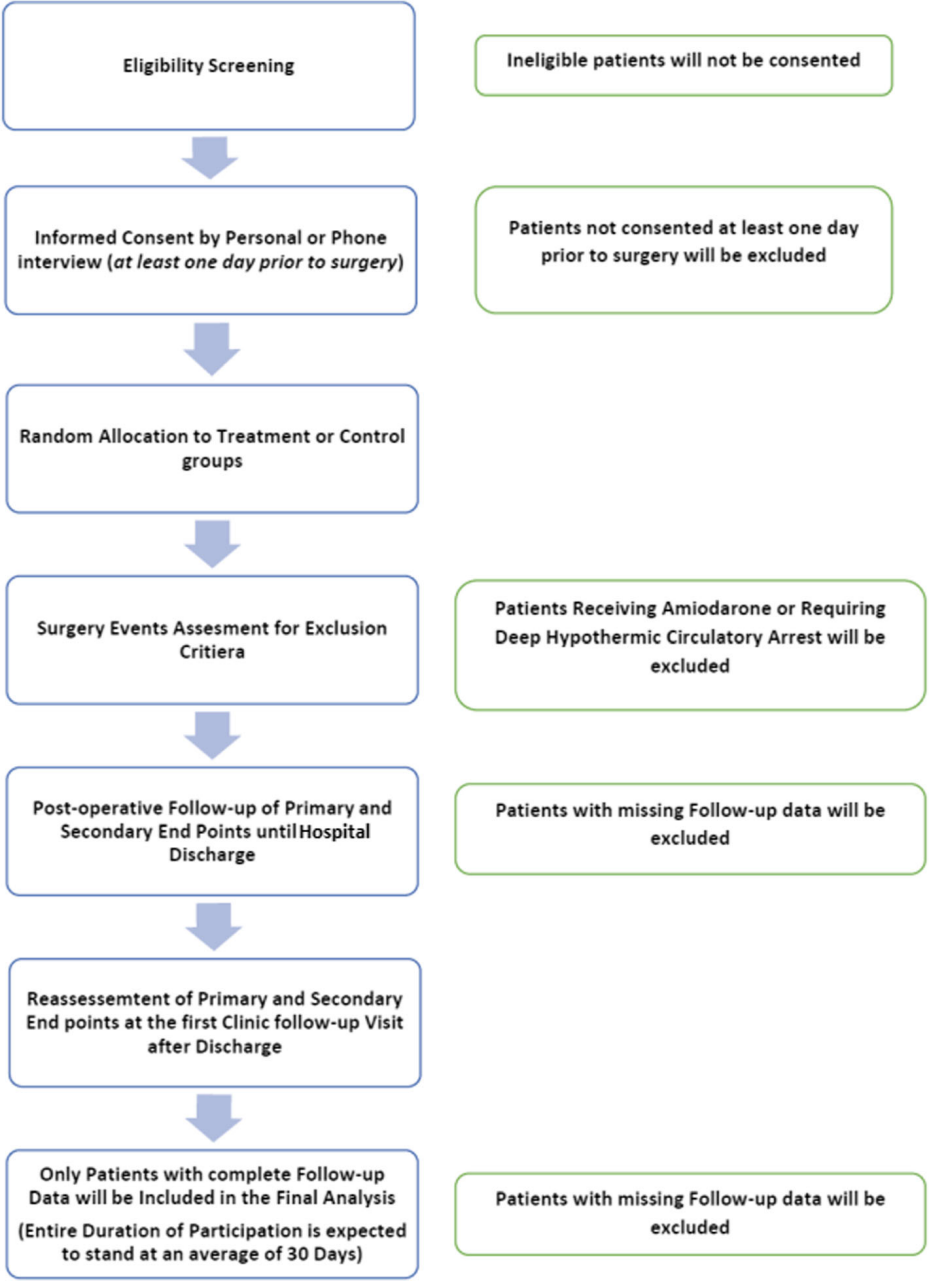
Participant timeline diagram: sequenced events of the trial modified from the Standard Protocol Items: Recommendations for Interventional Trials (SPIRIT) Statement

The main outcome of interest is the occurrence of POAF after cardiac surgery. Patients will first be screened for eligibility and willingness to participate in the trial. Then, informed consent will be collected from participating patients, who will be randomized into one of the two study groups. Postoperatively, patients will be closely monitored for the occurrence of atrial fibrillation, pleural and pericardial effusions. Times of event and to discharge will also be assessed. These outcomes will be monitored until the time of the follow-up visit, which will be within 30 days postoperatively for the majority of patients at our institution. Participation until this time point represents complete participation in the trial. We intend to include only patients who have completed follow-up (Fig. 2).

#### Sample size

Two groups are necessary for this trial: the control group and the intervention group. Based on analysis performed on our aortic surgery database for the years 2012 and 2013, the incidence of POAF in cardiac surgery patients was calculated to be 37.6%. A thorough study of the academic literature, including a meta-analysis of similar studies [10] shows that intervention reduces atrial fibrillation incidence by 50%. Therefore, an atrial fibrillation incidence of 40% in the control group with a proposed decrease of 50% with intervention requires a total of 158 subjects at 80% power and 0.05 alpha. Should the incidence of atrial fibrillation be 30% in the control with a hypothesized 50% decrease, a total of 322 subjects would be required at 90% power. In order to remain conservative, account for 5% protocol violation and 10% loss to follow-up/dropout, and retain power, a total sample size of 350 (175 patients in each treatment group) will be sufficient for this study. Should the incidence of POAF be lower than expected at interim analysis, sample size calculations will be re-estimated to ensure sufficient power at the time of analysis.

#### Recruitment

All consecutive patients admitted to the Department of Cardiothoracic Surgery at Weill Cornell Medicine/New York Presbyterian Hospital will be screened for enrollment. Patients will be enrolled by attending physicians, nurse practitioners, or the department research fellow. Included subjects will be patients undergoing open cardiac surgery for intervention on the coronary arteries, the aortic valve, and/or the ascending aorta, who have no previous history of atrial fibrillation. Estimated duration of accrual is 18 months with an expected 30-day duration for individual patients. We anticipate that at our high-volume center we will be able to recruit the needed 350 patients within 2 years. Patients are expected to stay enrolled in the trial until their single postoperative follow-up visit, which is usually within 30 days.

### Methods: assignment of interventions

#### Allocation

Allocation to treatment groups will be done in a controlled, randomized fashion. In order to assure an equal distribution of cases at different risk of POAF in the two groups after enrollment, the CHADS2 score, which has been shown to predict POAF in cardiac surgery patients, will be calculated [11]. Patients will be assigned to a lower risk (CHADS2Vasc score ≤ 2) or higher risk (CHADS2Vasc score ≥ 3). The cases will, therefore, be stratified based on the CHADS2Vasc score. Subsequently, a computer-generated, mixed block randomization will be performed in order to determine assignment between the control group and the intervention group. Randomly selected block sizes of 4, 6, and 8 will ensure stratification of risk levels in each treatment. At the time of pre-surgery time-out, the circulating nurse will open a sealed envelope and reveal the group to which the patient has been assigned.

#### Blinding (masking)

**Fig. 3:**
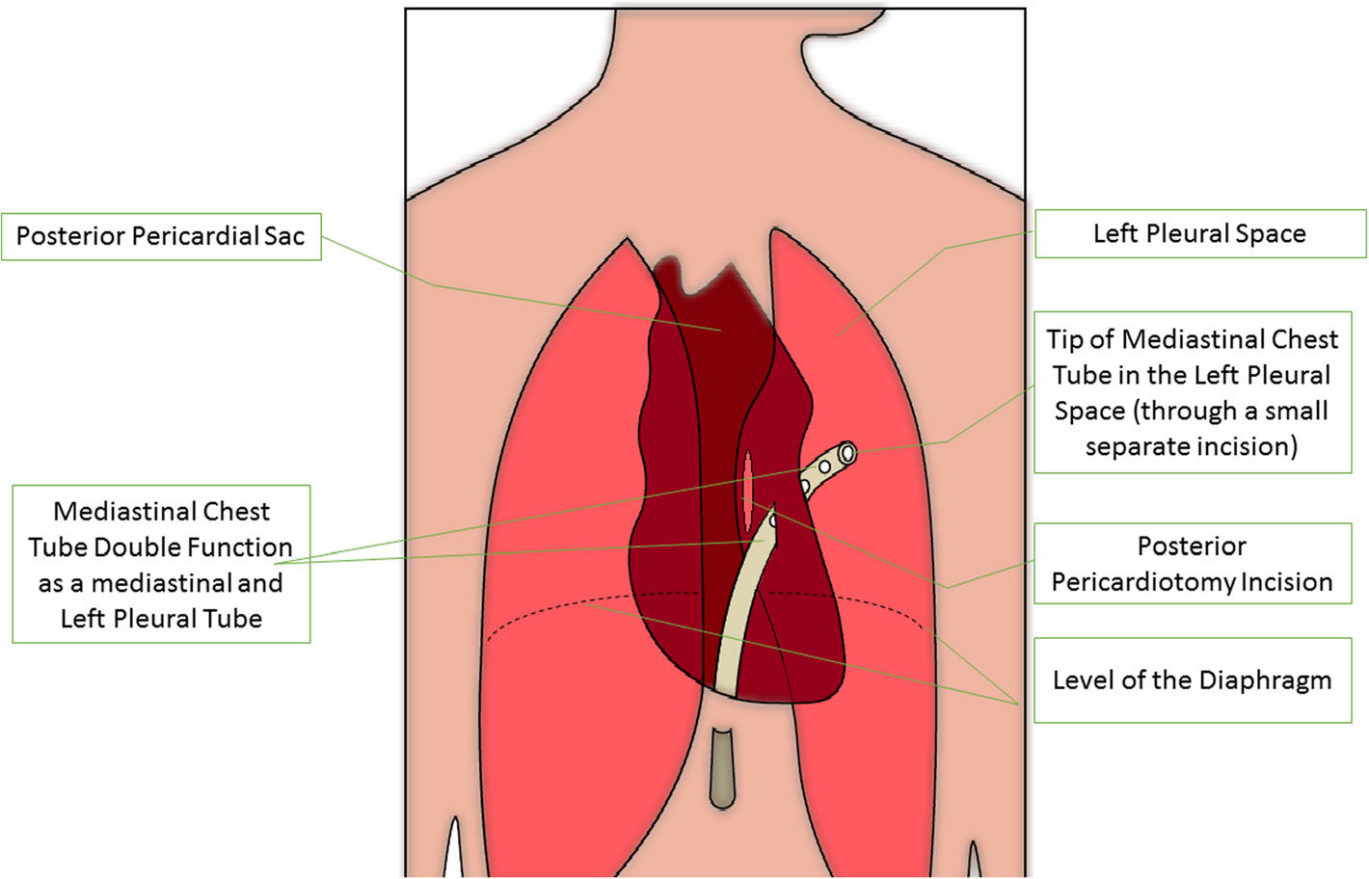
Double function of the mediastinal chest tube in posterior pericardiotomy patients

The hypothesis of blinding was considered during the study design. However, posterior pericardiotomy requires opening of the left-sided pleura and positioning of the tip of the mediastinal tube in the left-sided thorax so that patients in the intervention group will be distinguishable from those in the control group who may or may not have a left-sided pleural tube in situ. For those patients in the intervention group, the standard mediastinal chest tube will serve doubly functional as a mediastinal tube and a left-sided pleural tube (Fig. 3). This will lead to a single-blinded study, with the patients not knowing to which arm of the study they are assigned. This temporary suspension will be lifted after follow-up is completed. Premature unblinding will only be permitted if secondary interventions are required for treatment of complications related to the primary intervention.

All of the patients in the study will have a pre-bypass and post-bypass transesophageal echocardiographic (TEE) examination performed. Pre-operative and perioperative diastolic dysfunction in cardiac patients has been shown to be predictor of poorer outcomes. Our primary objective is to evaluate whether or not worsening right ventricular (RV) systolic or diastolic function affects the rate of POAF. Additionally, we hypothesize that the posterior pericardiotomy may affect atrial filling, as measured by pulmonary venous flow and left atrial contraction indices and the diastolic filling patterns of the left ventricle (LV).

Echo images will be taken before sternotomy and after chest closure to assess for left and RV diastolic dysfunction, and well as left atrial filling indices. These images will be taken as part of the routine TEE examination, but at the time points specified above to minimize acute hemodynamic and volume changes.

Specific measurements to be recorded are the transmitral flow (mitral E and A waves, A duration, deceleration time), pulmonary venous flow (S and D waves and A duration), left atrial appendage velocities, tissue Doppler of the LV wall (medial and lateral e’), the RV fractional area of change (FAC), the hepatic venous flow (S, D, and A waves), and the transtricupsid flow (tricuspid E and A waves). Post-processing measurements will also be taken of RV and LV strain to assess whether a change in strain is predictive of POAF. Echocardiographers will be blinded to the intervention unless there is a complication that requires anesthetic intervention that is unique to undergoing posterior pericardiotomy.

### Methods: data collection methods

#### Data collection methods

Data will be prospectively collected from the time of enrollment and during the entire hospital stay by filling the dedicated Data Sheet Form. The principal investigator, statistician, and research fellow will have access to the final data sets; however, all participating researchers will have access to view the final data. Data will be entered into the database and monitored daily for quality and accuracy by a dedicated research fellow.

#### Retention

Before enrollment and prior to discharge, patients will be reminded and encouraged to comply with follow-up. Patients who deviate from follow-up will be contacted by phone and encouraged to complete follow-up.

#### Data management

##### Statistical methods

Pre-operative differences between the control and the intervention group will be assessed by univariate analysis. Continuous variables will be analyzed by way of Student’s *t* test or the Mann-Whitney *U* test and categorical variables will be analyzed by use of the chi-squared test. Postoperative differences, including atrial fibrillation and other complications, will also be assessed between the control and intervention groups by way of univariate analysis.

##### Outcomes

The primary outcome of the study is the occurrence of POAF, defined as the occurrence of an irregularly irregular heart rhythm, without detectable P waves, lasting more than 30 s observed during the hospital stay. Patients will be monitored continuously during the entire hospital stay with the Philips Intellivue MP70 patient monitor (Philips, Andover, MA, USA) and alarm strips will be collected for analysis and POAF adjudication. A standard 12-lead electrocardiogram (EKG) will be recorded on a daily basis and collected for analysis and POAF adjudication. Additional 12-lead standard EKGs ordered at the discretion of the caring physician will be collected for analysis and POAF adjudication. An independent committee blinded to group assignment will adjudicate episodes of POAF. The secondary outcome measures of the study will include: (1) time spent in atrial fibrillation, defined as the time from the first evidence of atrial fibrillation to the first evidence of sinus rhythm restoration on cardiac monitoring strips or standard EKG, (2) duration of hospitalization, (3) antiarrhythmic drug use, (4) need for electrical cardioversion. Safety outcomes will be (1) incidence of left-sided pleural effusion, (2) incidence of pericardial effusion, (3) MAE, and (4) death.

##### Additional analyses

The primary outcomes will be tested according to the intention-to-treat principle (ITT) where all participants will be included in their assigned treatment groups regardless of the actual procedure performed. Modified intention-to-treat analysis will also be conducted. Per-protocol or as-treated analysis will also be reported as descriptive. The primary outcome analysis will be assessed by logistic regression. Baseline covariates will be included in the regression model for sensitivity analysis. These covariates include: age, sex, diabetes status, ejection fraction, extent of coronary disease, on or off pump procedure, surgical priority, and completeness of revascularization. Secondary outcomes will be analyzed for safety by descriptive statistics.

##### Analysis population and missing data

Authors do not expect missing data since data collection is during a short period for each patient and only one follow-up visit. In rare instances of missing data, multiple imputation analysis will be utilized. The loss to follow-up will also be reported and compared between the groups using absolute risk differences.

### Methods: monitoring

#### Data monitoring

##### Formal committee

A Data Monitoring Committee (DMC) has been established. The DMC is composed of two cardiologists, one cardiac surgeon, and one cardiac anesthesiologist who are not involved in the study and not participating in the care of enrolled patients. Reports will be made available after every 50 patients that are accrued.

##### Interim analyses

Interim analyses will be performed after enrollment of the first and second 100 consecutive patients. Results from interim analyses will be reported to the DMC and the medical monitor (a cardiac anesthesiologist not participating in the study or the care of the patients). The principal investigator (PI) will be blinded to these results. Using the Haybittle-Peto rule for efficacy analysis, a difference of at least four standard deviations for the first interim analysis and three standard deviations at the interim analysis in the incidence of the primary outcome will justify premature halting of the study. In order to be considered significant, the corresponding chi-square value is 16 (*α* = 0.001).

##### Harms

Serious adverse events (SAE) will be reported to the DMC, the Institutional Review Board and the PI. The study will be interrupted in case of significant differences between groups at interim analyses in terms of SAE during the hospitalization. In cases of mortality, if it is determined that death was a direct result of the trial intervention, the study will be stopped. A subject may be removed from the study if, during the time of surgery, the attending surgeon determines that it would be unsafe to perform the posterior left pericardiotomy due to unexpected difficulty during surgery, additional cardiac surgery, extended cardiac ischemic time, or hemodynamic instability. Removed patients will continue to receive the same care had they not been enrolled. The removed patient will be replaced in the trial should this occur before randomization. The results of the patient will not be included in the dataset or analysis. Since the primary analysis is based on ITT then the patient will be included in the analysis if they are already randomized.

### Methods: ethics and dissemination

#### Research ethics approval

The study was approved by the Institutional Review Board on 26 May 2016: Protocol number 1502015867R001.

#### Consent or assent

Consent is required prior to individual patient enrollment according to institutional guidelines. Research will be performed according to the Declaration of Helsinki.

#### Ancillary studies

All of the patients in the study will have a pre-bypass and post-bypass TEE examination performed. Pre-operative and perioperative diastolic dysfunction in cardiac patients has been shown to be predictor of poorer outcomes. Ashes et al. demonstrated a higher incidence of POAF in CABG patients with worsening diastolic function perioperatively compared to patients with unchanged or improved diastolic function [12]. Other studies have shown that pre-operative diastolic dysfunction predicted higher incidence of POAF in cardiac surgical patients. Our primary objective is to evaluate whether or not worsening RV systolic or diastolic function affects the rate of POAF. Additionally, we hypothesize that the posterior pericardiotomy may affect atrial filling, as measured by the pulmonary venous flow and left atrial contraction indices and the diastolic filling patterns of the LV.

Echo images will be taken before sternotomy and after chest closure to assess for left and RV diastolic dysfunction, and well as left atrial filling indices. These images will be taken as part of the routine TEE examination, but at the time points specified above to minimize acute hemodynamic and volume changes.

Specific measurements to be recorded are the transmitral flow (mitral E and A waves, A duration, deceleration time), pulmonary venous flow (S and D waves and A duration), left atrial appendage velocities, tissue Doppler of the LV wall (medial and lateral e’), the RV fractional area of change (FAC), the hepatic venous flow (S, D, and A waves), and the transtricupsid flow (tricuspid E and A waves). These measurements will be taken offline and are part of the standard examination and American Society of Echocardiography (ASE) guidelines on quantification of LV diastolic dysfunction [13, 14]. Post-processing measurements will also be taken of RV and LV strain to assess whether a change in strain is predictive of POAF. Echocardiographers will be blinded to the intervention unless there is a complication that requires anesthetic intervention that is unique to having a posterior pericardiotomy.

#### Confidentiality

The data will be de-identified upon entry into the data base.

#### Access to data

Data will be stored using Microsoft Access 2010 software (Microsoft, Redmond, WA, USA) and analyzed using IBM SPSS Statistics version 22 (IBM, Armonk, NY, USA) All networks, emails, and computers used for the analysis are institutional and protected by individual login requirements along with a team of cyber security personnel.

#### Dissemination policy

##### Trial results

Data that break the blinding of the study will not be presented prior to the release of main results. Final results of the study will be reported in a manuscript and submitted to a peer-reviewed journal.

##### Authorship

The study group will follow the criteria of The International Committee of Medical Journal Editors to grant authorship for manuscripts submitted for publication [15].

##### Reproducible research

Public access to the full protocol, de-identified, participant-level dataset access, and statistical code will be allowed 5 years after the main results’ publication.

## Discussion

POAF has continued to be a source of major morbidity, prolongation of hospitalization and additional costs following cardiac surgery. In the surgical literature, an average of one additional hospital day with a burden of about €1800 per patient has been reported. Early treatment was associated with a lower incidence of cerebrovascular events [16]. In another risk-adjusted study, POAF resulted in a twofold increase in mortality (adjusted odds ratio = 2.04, *p* < 0.001), two additional intensive care unit (ICU) days (*p* < 0.001), three additional hospital days (*p* < 0.001), and $3000 (*p* < 0.001) and $9000 (*p* < 0.001) of increased ICU and total hospital-related costs, respectively [17]. Although numerous methods and treatment options have been examined over the years, none has proven to be optimal. Currently, pharmacological agents are the most often used modality for the prevention and treatment of POAF. While some of these agents may help reduce POAF, their side effects and costs cannot be ignored. Thus, a cost-effective and a low-risk intervention for POAF is yet to be provided. This topic is of great interest to the cardiovascular community and the results of this study could have profound implications on current practices. The potential to decrease POAF and its associated complications will improve our clinical knowledge and surgical practice while decreasing patient morbidity and mortality. Similar to any surgical procedure, posterior pericardiotomy is associated with potential complications. Aside from the complications associated with any cardiac procedure, it is associated with a risk of phrenic nerve injury, cardiac herniation, and extended drainage time of left-sided pleural effusions. These complications can be prevented by careful technique and postoperative management. Proper operative identification of the phrenic nerve should be emphasized. In addition, special attention should be given to not extend the posterior pericardiotomy incision beyond 4 cm to avoid the risk of cardiac herniation [6].

In a previous meta-analysis by Kaleda et al., posterior pericardiotomy was found to significantly reduce the incidence of POAF. This study showed a substantial reduction in total arrhythmias and atrial fibrillation in the posterior pericardiotomy group (odds ratio 0.31 and 0.33, respectively). The reported number needed to treat was six patients to prevent one case of atrial fibrillation. However, this meta-analysis was non-conclusive as it was based on seven studies which were either cohort or non-blinded studies. In addition, the outcomes assessed were not homogenous, with primary outcome measures varying between the incidence of postoperative pericardial effusions, arrhythmias or atrial fibrillation [6]. In 1999, Kurlay et al. reported the results of a randomized controlled trial. In 200 patients, atrial fibrillation rate (6%) was significantly lower in the posterior pericardiotomy group in comparison with the control group (34%) (*p* < 0.001). In the control group, early and late pericardial effusion rates were 54% and 21%, respectively, in contrast to that of the posterior pericardiotomy group which was zero for both (*p* < 0.001). Delayed pericardial tamponade was also significantly lower in group I (0% vs. 10%; *p* = 0.001). However, in this trial, patients receiving beta-blockers were excluded and, therefore, its results are not relevant to current practice [7]. In a more recent randomized trial, similar methodology was utilized with promising results in 50 posterior pericardiotomy patients. POAF was significantly lower in the posterior pericardiotomy group compared with the control group (10% vs. 30%, *p* < 0.010). Perioperative pericardial effusion rate was 12% compared to 42% in the control group (*p* < 0.001). The overall incidence of total arrhythmias in patients with early pericardial effusion was significantly higher than in those without this complication (18 vs. 9 patients) [8].

The present randomized study should significantly contribute to the assessment of the role of posterior pericardiotomy to reduce the incidence of atrial fibrillation after cardiac surgery.

### Trial status

This trial was registered at ClinicalTrials.gov in July 2016 with protocol record 1502015867 and identifier NCT02875405.

The date of initial recruitment: 1 August 2017.

The approximate date when recruitment will be completed: 1 August 2018.

## Additional file

Additional file 1: SPIRIT Checklist, Figure S1: SPIRIT Checklist. (DOC 124 kb)

## Abbreviations

BPM: Beats per minute
CXR: Chest radiograph
DMC: Data Monitoring Committee
EKG: Electrocardiogram
FAC: Fractional area of change
ITT: Intention-to-treat principle
MAE: Major adverse events
PI: Principal investigator
POAF: Postoperative atrial fibrillation
