# Supplementary material for: Posterior Left pericardiotomy for the prevention of postoperative Atrial fibrillation after Cardiac Surgery (PALACS): study protocol for a randomized controlled trial
Source: Trials. 2017 Dec 13;18:593. doi: 10.1186/s13063-017-2334-4 (PMC5729517; doi:10.1186/s13063-017-2334-4)
Supplement: Additional file 1: — SPIRIT Checklist, Figure S1: SPIRIT Checklist. (DOC 124 kb) [file 13063_2017_2334_MOESM1_ESM.doc]

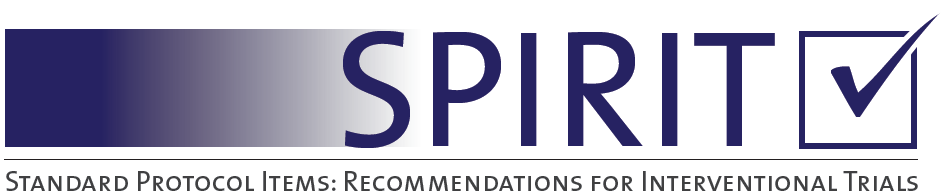


SPIRIT 2013 Checklist: Recommended items to address in a clinical trial protocol and related documents*

| Section/item | Item No | Description |
| --- | --- | --- |
| **Administrative information** | | |
| Title | 1 | Posterior Left Pericardiotomy for the Prevention of Postoperative Atrial Fibrillation after Cardiac Surgery (PALACS): study protocol for a randomized controlled trial |
| Trial registration | 2a | This trial was registered at clinicaltrials.gov in July 2016. Protocol record 1502015867 and identifier NCT02875405. |
| Protocol version | 3 | 08/22/2016, protocol record 1502015867 and identifier NCT02875405. |
| Funding | 4 | No external funding to declare. |
| Roles and responsibilities | 5a | **AAA, JRL** were responsible for assessing eligibility for enrolment, consenting eligible patients, collecting rhythm strips, follow up data, management of data sheets, and trial updates.  **LBO** contributed by performing the literature review, writing the manuscript, intellectual analysis of manuscript, read and approved the final manuscript.  **CL** was integral to revision and editing manuscript, intellectual analysis of manuscript, read and approved the final manuscript.  **MM** contributed by performing statistical analysis for the trial.  FC, MM contributed by performing review of the collected rhythm strips for confirmation of Atrial Fibrillation.  **TS** helped create the study design, reviewed content and intellectual analysis of the manuscript, and read and approved the final manuscript.  **KOP, NSI** was involved and contributed to conceptual design of the ancillary study, critical review for intellectual content, read and approved the final manuscript.  **LQR** was involved and contributed to conceptual design of the ancillary study, critical review for intellectual content, read and approved the final manuscript and was responsible for the pre and post-operative Echo performance and generation.  **LNG** was involved and contributed to conceptual design, critical review for intellectual content, read and approved the final manuscript.  **MG** was integral to creation of the study design, helped review content, participated in writing the manuscript and verifying the intellectual analysis of the manuscript, read and approved the final manuscript.  Department of Cardiothoracic Surgery, Weill Cornell Medicine, New York, New York United States.  1Department of Cardiovascular Sciences, Catholic University, Rome, Italy |
|  | 5b | Weill Cornell Medicine Cardiothoracic Surgery  525 East 68th Street, Box 110, Suite M 404, New York, NY 10065  Cardiac Surgery: (212) 746-5194  Email: ctsurgery@med.cornell.edu |
|  | 5c | N/A |
|  | 5d | External collaboration will be done with two cardiologists and one cardiac surgeon from the Catholic University, Rome Italy. All these individuals have considerable experience in the field and have performed extensive research on this topic. Rhythm strips in cases of suspected POAF will be sent for a blinded interpretation. Their blinded judgement will assure objective evaluation of the EKG data. Study design and execution will also be collaborated when necessary. |
| Introduction |  |  |
| Background and rationale | 6a | Post-operative atrial fibrillation (POAF) is a common complication following cardiac surgery. POAF is associated with increased morbidity and hospital costs. We herein describe the protocol for a randomized control trial to determine if performing a posterior left pericardiotomy prevents POAF after cardiac surgery. |
|  | 6b | Allocation to treatment groups will be done in a controlled randomized fashion. In order to assure an equal distribution of cases at different risk of POAF in the two groups after enrolment. The CHADS2 score, which has been shown to predict POAF in cardiac surgery patients, will be calculated [*J Thorac Cardiovasc Surg. 2013 Oct; 146(4):919–926.e1*]. |
| Objectives | 7 | The primary objective of the present prospective, randomized-controlled study is to assess whether performing a posterior left pericardiotomy during open cardiac surgery procedures results in a reduction in the incidence of POAF.  The secondary outcome measures of the study will include: (a) time spent in atrial fibrillation, defined as the time from the first evidence of atrial fibrillation to the first evidence of sinus rhythm restoration on cardiac monitoring strips or standard EKG; (b) duration of hospitalization; (c) antiarrhythmic drug use; (d) need for electrical cardioversion. Safety outcomes will be 1) incidence of left pleural effusion; 2) incidence of pericardial effusion 3) MAE and 4) death. |
| Trial design | 8 | Allocation to treatment groups will be done in a controlled randomized fashion. The cases would therefore be stratified based on the CHADS2vasc score. Subsequently, a computer generated four block randomization would be performed in order to determine assignment between the control group and the intervention group. |
| Methods: Participants, interventions, and outcomes | | |
| Study setting | 9 | This study will be conducted in Weill Cornell Medicine/New York Presbyterian Hospital. |
| Eligibility criteria | 10 | All consecutive patients admitted to the Department of Cardiothoracic Surgery will be screened for enrolment. Included subjects will be patients undergoing open cardiac surgery for interventions on coronary arteries, aortic valve, ascending aorta, and who have no previous history of atrial fibrillation. Exclusion criteria are as follows: preoperative non sinus rhythm, history of previous atrial arrhythmia of any type, reoperation, mitral or tricuspid valve disease, surgery of the descending thoracic or thoracoabdominal aorta, need for hypothermic circulatory arrest, urgent or emergent presentation, disease of the left pleura or previous left sided instrumentation, non-cardiac related comorbid contraindications to surgery, and chest deformity of any kind. |
| Interventions | 11a | In the intervention group, a posterior left pericardiotomy will be performed according to technique described in the literature [*Eur J Cardio-Thorac Surg off J Eur Assoc Cardio-Thorac Surg. 1995; 9(3):150–2*.]. In brief, if not already entered the left plural space will be entered through the pre-existing median sternotomy. A 4 cm incision will be made posterior to the phrenic nerve in a parallel and longitudinal fashion extending from the left inferior pulmonary vein to the diaphragm. We will use the tip of the mediastinal tube that is placed in all cardiac surgery patients (instead of an additional chest tube) for drainage of the left pleura so that patients of the treatment group will not have a higher level of postoperative pain and discomfort. The estimated additional surgery time is 15 minutes. |
|  | 11b | Interim analyses will be performed after enrollment of the first and second 100 consecutive patients. |
|  | 11c | Results from interim analyses will be reported to the DCM, the Principal Investigator (PI) and the medical monitor (a cardiac anaesthesiologist not participating in the study or the care of the patients). Using the Haybittle-Peto rule, a difference of at least four standard deviations for the first interim analysis and three standard deviations at the interim analysis in the incidence of the primary outcome will justify premature halting of the study. In order to be considered significant, the corresponding chi-square value is 16 (α=0.001). |
| Outcomes | 12 | The primary end-point of the study is the occurrence of POAF, defined as the occurrence of irregular heart rhythm, without detectable P-waves, lasting more than 30 seconds observed during the hospital stay after open cardiac surgery.  The secondary end-points of the study are: (a) time spent in atrial fibrillation; (b) duration of hospitalization; (c) antiarrhythmic drug use; (d) need for electrical cardioversion.  Safety outcomes will be; 1) incidence of left pleural effusion; 2) incidence of pericardial effusion 3) MAE and 4) death. |
| Participant timeline | 13 | 30 Days. |
| Sample size | 14 | Two groups are necessary for this trial: the control group and the intervention group. Based on analysis performed on our aortic surgery database for the years 2012 and 2013, the incidence of postoperative atrial fibrillation in cardiac surgery patients was calculated to be 37.6%. A thorough study of the literature, shows that intervention reduces atrial fibrillation incidence by 50%. Therefore, an atrial fibrillation incidence of 40% in the control group with a proposed decrease of 50% with intervention requires a total of 158 subjects at 80% power and 0.05 alpha. Should the incidence of atrial fibrillation be 30% in the control with a hypothesized 50% decrease, a total of 322 subjects would be required at 90% power. In order to remain conservative, account for 5% protocol violation and 10% loss to follow-up/dropouts, and retain power, a total sample size of 350 (175 patients in each treatment group) will be sufficient for this study. Should the incidence of POAF be lower than expected when 350 patients are enrolled, 50 events in the control group will be included in place of sample size to ensure sufficient power at the time of analysis. |
| Recruitment | 15 | Wide range of surgeries including coronary, aortic valve and ascending aortic surgeries will be asked for participation. |
| **Methods: Assignment of interventions (for controlled trials)** | | |
| Allocation: |  |  |
| Sequence generation | 16a | Patients will be assigned to a lower risk (CHADS2Vasc score ≤2) or higher risk (CHADS2Vasc score ≥3). Subsequently, a computer generated four block randomization would be performed in order to determine assignment between the control group and the intervention group. |
| Allocation concealment mechanism | 16b | At the time of pre-surgery time-out, the circulating nurse will open a sealed envelope and reveal the group to whom the patient has been assigned. |
| Implementation | 16c | Computer generated four block randomization would be performed in order to determine assignment between the control group and the intervention group. Data will be entered into the database and monitored daily for quality and accuracy by a dedicated research fellow. |
| Blinding (masking) | 17a | Posterior pericardiotomy requires opening of the left pleura and positioning of the tip of the mediastinal tube in the left thorax so that patients in the intervention group will be distinguishable from those in the control group. This will lead to a single blinded study, with the patients not knowing to which arm of the study they are assigned. This temporary suspension will be lifted after follow-up is completed. Premature unblinding will only be permitted if secondary interventions are required for treatment of complications related to the primary intervention. |
|  | 17b | This temporary suspension will be lifted after follow-up is completed. Premature unblinding will only be permitted if secondary interventions are required for treatment of complications related to the primary intervention. |
| **Methods: Data collection, management, and analysis** | | |
| Data collection methods | 18a | Data will be prospectively collected from the time of enrolment and during the entire hospital stay by filling the dedicated data sheet form. |
|  | 18b | Data will be entered into the database and monitored daily for quality and accuracy by a dedicated research fellow. |
| Data management | 19 | The principle investigator, statistician, and research fellow will have access to the final data sets, however, all participating researchers will have access to view the final data. Data will be de-identified and kept in a secure server. |
| Statistical methods | 20a | Preoperative differences will be assessed by univariate analysis. Continuous variables will be analysed by student’s t-test or Mann-Whitney U and categorical variables will be analyzed by the chi-squared test. Postoperative differences, will also be assessed by univariate analysis. Logistic regression analysis will be performed in order to assess for significant predictors of POAF or other postoperative variables that may be significant. |
| Methods: Monitoring | | |
| Data monitoring | 21a | A Data Safety Monitoring Board will be used and composed of two cardiologists and one cardiac surgeon. They will be part of an external collaboration with the Catholic University of Rome. Our institutional medical monitor will be a cardiac anaesthesiologist who is not part of the study and not participating in the care of enrolled patients. Reports will be made available after every 50 patients that are accrued. |
| Harms | 22 | Serious adverse events (SAE) will be reported to the DCM, the Institutional Review Board and the PI. The study will be interrupted in case of significant differences between groups at interim analyses in terms of SAE during the hospitalization. In cases of mortality, if it is determined that death was a direct result of the trial intervention, the study will be stopped. A subject may be removed from the study if, during the time of surgery, the attending surgeon determines that it would be unsafe to perform the posterior left pericardiotomy due to unexpected difficulty during surgery, additional cardiac surgery, extended cardiac ischemic time, or hemodynamic instability. Removed patients will continue to receive the same care had they not been enrolled |
| Auditing | 23 | Reports will be made available after every 50 patients that are accrued. |
| Ethics and dissemination | | |
| Research ethics approval | 24 | The study was approved the Institutional Review Board on May 26th 2016. Protocol number 1502015867R001. Consent is required prior to individual patient enrolment according to institutional guidelines. Research will be performed according to the Declaration of Helsinki. |
| Protocol amendments | 25 | Updates will be made and reports every 50 patients that are accrued. |
| Consent or assent | 26a | A dedicated research fellow will consent patients after comprehensive explanation of the study and roles using a consent form. |
| Confidentiality | 27 | The data will be de-identified upon entry into the data base. All networks, emails, and computers used for the analysis are institutional and protected by individual login requirements along with a team of cyber security personal. Results from interim analysis will be reported to the principle investigator and the medical monitor. Serious adverse events will be reported to the institutional review board. |
| Declaration of interests | 28 | The authors declare that they have no competing interests. |
| Access to data | 29 | The principle investigator, statistician, and research fellow will have access to the final data sets, however, all participating researchers will have access to view the final data. |
| Ancillary and post-trial care | 30 | N/A |
| Dissemination policy | 31a | This trial was registered at clinicaltrials.gov in July 2016. Protocol record 1502015867 and identifier NCT02875405. |
|  | 31b | N/A |
|  | 31c | This trial was registered at clinicaltrials.gov in July 2016. Protocol record 1502015867 and identifier NCT02875405. Its status is Public, awaiting imminent recruitment initiation. |
| Appendices |  |  |
| Informed consent materials | 32 | Model consent form and other related documentation given to participants and authorised surrogates |
| Biological specimens | 33 | Plans for collection, laboratory evaluation, and storage of biological specimens for genetic or molecular analysis in the current trial and for future use in ancillary studies, if applicable |

*It is strongly recommended that this checklist be read in conjunction with the SPIRIT 2013 Explanation & Elaboration for important clarification on the items. Amendments to the protocol should be tracked and dated. The SPIRIT checklist is copyrighted by the SPIRIT Group under the Creative Commons “[Attribution-NonCommercial-NoDerivs 3.0 Unported](http://www.creativecommons.org/licenses/by-nc-nd/3.0/)” license.
